# A rare case of 46, XX (SRY positive) testicular disorder of sex development with growth hormone deficiency: Case report

**DOI:** 10.1097/MD.0000000000024641

**Published:** 2021-02-12

**Authors:** Hanming Li, Jianyu He, Iatlun Leong

**Affiliations:** a Pediatrics of the Fifth People's Hospital of Foshan City, Guangdong; b General Surgery of University Hospital of Macau SAR, China.

**Keywords:** 46, 46, growth hormone deficiency, testicular disorder of sex development, whole exome gene sequencing, XX (SRY positive), XX male syndrome

## Abstract

**Rationale:**

Chromosome karyotype analysis and SRY (sex determined region of Y chromosome) gene detection are routines for the diagnosis of growth hormone deficiency (GHD), but further whole exome gene sequencing occasionally leads to subversive results and unexpected conclusions.

**Patient concerns:**

We report a single case of a 7-year-old Chinese boy who had stunted growth since he was 1 year old. He was short in height (height Standard Deviation Score (SDS) was less than 2.9), bilateral scrotal dysplasia and delayed bone age.

**Diagnosis:**

His growth hormone (GH) stimulation tests showed GHD. His karyotype analysis and polymerase chain reaction (PCR) analyses indicated a 46, XX disorder of sex development (DSD) without the presence of the SRY gene. Nevertheless, considering that female gonad was not observed in the chest and abdominal magnetic resonance imaging, the whole exome gene sequencing was performed. Sequencing data confirmed the presence of SRY gene sequence and two copies of chromosome X. Later, using different primer sequences for PCR, it showed that the SRY gene was positive. The final diagnosis was a rare case of “46, XX (SRY positive) testicular DSD with GHD”.

**Interventions:**

The boy's parents agreed to use recombinant human growth hormone (rhGH) for GHD treatment, the starting dose was 0.035 mg / kg / day. But they disagreed with molecular diagnostics and genomic analysis of the Y chromosome.

**Outcomes:**

The boy was treated with rhGH for 3 months and his height increased by 2.2 cm. The patient will be followed-up until the end of his puberty.

**Lessons:**

In summary, whole exome gene sequencing overturned the preliminary diagnosis results of karyotype analysis and SRY gene detection, and found that there may be a certain correlation between testicular DSD and GHD.

## 1 Introduction

In 1990, Sinclair et al suggested that the SRY gene located on chromosome Y (Yp11.32) could be a candidate for the elusive testis-determining gene.^[1]^ Previous studies have indicated that deletion, mutation, translocation, and inversion duplication of the SRY gene may lead to DSD.^[2]^ A 46, XX testicular disorder of sex development, is a human sex chromosomal aberration characterized by the inconsistency between chromosome and gonad, including 46, XX (SRY positive) and 46, XX (SRY negative). It is very rare and is estimated to occur in 1/20,000 newborn males^[3]^ which is characterized by infertility, feminized breasts, small testicle, only 20% of children with testicular DSD have an external genital malformation at birth. Compared to an adult stage, testicular DSD can be challenging to detect the disease at an early developmental stage.

The pathogenesis of 46, XX testicular DSD is still unclear. So far, few theories have been proposed:

1. Yp-Xp translocation hypothesis: In recent years, fluorescent in situ hybridization technology combined with SRY gene-specific probes has proven that there is a SRY gene probe signal at the distal end of the paternal-derived X chromosome short arm (Xp) in 46, XX male individuals.^[4]^ This result suggests that the translocation of the SRY gene to the X chromosome is a key factor leading to the development of 46, XX males into complete male sexual characteristics. A few other case studies reported that the SRY gene translocated to the long arm of X chromosome (Xq28).
2. Target gene mutation hypothesis: Studies suggested that SRY gene determines male sex differentiation by double regulation of inhibition on X chromosome and activation on Y chromosome, which makes SRY gene inhibit the expression of an autosomal gene, and makes structural genes spontaneously start up to 46, XX male without SRY gene activation.^[5]^ Currently, it is believed that DAX1, SF1, WNT4, WT1 and NR5A1 may also participate in the process of sex determination.^[6,7]^ These genes mainly play a role by changing the protein level or protein function in the process of sex determination.
3. SOX9 gene overexpression theory: The related autosomal gene SOX9 is also known from loss-of-function mutations in mice and humans to be essential for Sertoli cell differentiation. Moreover, its abnormal expression in an XX gonad can lead to male development in the absence of SRY. In 2018, the Francis Crick Institute found that in vivo high-throughput chromatin accessibility techniques, transgenic assays, and genome editing revealed several novel gonadal regulatory elements in the 2-megabase gene desert upstream of SOX9. Although others are redundant, enhancer 13 (Enh13), a 557-base pair element located 565 kilobases 5’ from the transcriptional start site, is conserved and embedded within a 32.5-kilobase region whose deletion in humans is associated with XY sex reversal, suggesting that it is also critical in humans.^[8]^ Coincidentally, bioinformatic analysis identified three putative enhancers for SOX9 that responded to different combinations of testis-specific regulators. All three enhancers showed synergistic activity and together drive SOX9 in the testis, when duplicated or deleted, result in 46, XX or 46, XY sex reversal, respectively. These enhancers provide a hitherto missing link by which SRY activates SOX9 in humans, and establish SOX9 enhancer mutations as a significant cause of DSD.^[9]^

The clinical characteristics based on testicular DSD are different, and their pathogenesis involve different molecular cytogenetic abnormalities. Sometimes, it is not enough to rely on the diagnosis of cell karyotype analysis or PCR. Furthermore, in the diagnosis of short stature, the connection between testicular DSD and GHD has not been taken seriously. Herein, we report a rare case of a patient who was diagnosed with 46, XX (SRY positive) testicular DSD with GHD after undergoing the whole exome gene sequencing for the SRY gene.

## 2 Case presentation

A 6-year-old Chinese boy was brought in by his parents for clinical consultation due to growth retardation they observed over the course of the past 5 years. He was the older son (he has a healthy younger brother) born at full-term spontaneous labor (48 cm in length, 2.4 kg in weight and head circumference 42.5 cm at birth). The boy was small for gestational age (SGA).^[10]^ When he was one year old, his parents noticed that he was growing slower, since he was 74 cm long (-1 SDS Boys^[10]^) but had no other symptoms. His parents are healthy, but both have short stature. Family history revealed non-consanguinous marriage and no family genetic history was found.

### 2.1 Clinical findings

In March 2019, at the time of first visit he was 6 years old. The physical examination indicated the following: his height was 105.7 cm (-2.6 SDS Boys; -2.4 SDS Girls^[10]^) and BMI 14.1 kg / m^2^. No special facial appearance, coordinated body proportions, male genitalia, Tanner stage I, penis length of 2.0 cm (6-year-old boy reference range:4.14 ± 0.43 cm^[11]^), bilateral scrotal dysplasia, and no testicles in the scrotum. The urethral meatus was located distally on the penile shaft. His father and mother's height were 161 cm (-2 SDS^[10]^) and 150 cm (-2 SDS^[10]^), respectively. They both had normal sexual characteristics, and both were Tanner stage V.

**Timeline**

**Table:** 

Time	Important milestones
..March 2013	The patient was SGA
2014	The patient was found to grow slowly
March 2019	The time of first visit
April 2019	Discovery of GHD and SRY gene deletion
May 2019	Diagnosis of 46, XX (SRY positive) testicular DSD with GHD by whole exome gene sequencing
May 2020	GHD was confirmed again, rhGH treatment started
August 2020	Follow-up for 3 months after treatment, height increased by 2.2cm

### 2.2 Diagnostic assessment

**Figure 1:**
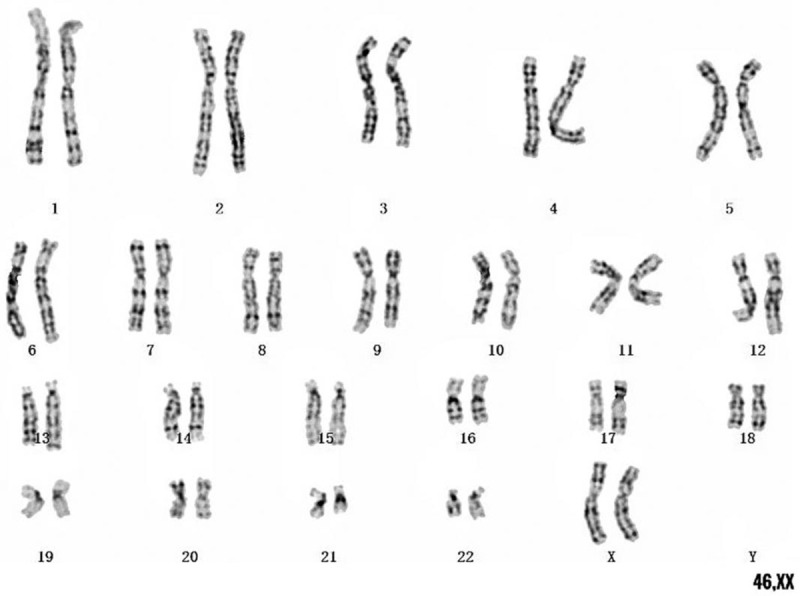
Results of karyotype analysis: 46, XX.

The boy's intelligence test (Wechsler-preschool and primary scale of Intelligence, WPPSI) was normal. The routine blood test, routine urine test were within the normal range. His testosterone was 0.19 ng/ml (reference range: <0.1–0.35 ng/ml), follicle-stimulating hormone was 0.90 IU/L (reference range: 0.26–3.0 IU/L), luteinizing hormone was 0.01 IU/L (reference range: < 0.3 IU/L), and thyroid hormones were all within normal levels. IGFBP3 and IGF-I levels were 3.36 μg/ml (-1.5 SDS 6-year-old Boys^[12]^) and 107.6 ng/ml (-1.3 SDS 6-year-old Boys^[12]^), respectively. Ultrasound examination showed that patient's bilateral gonads were pushed up 11 mm, and there was no adrenal and celiac ectopic hyperplastic disease. Besides, his bone age was delayed by 1 year (Greulich-Pyle Radiographic atlas of skeletal development of hand and wrist, Boys). Magnetic resonance imaging of the pituitary gland was normal. The peak level of the arginine stimulation test was 6.64ng/ml by Radioimmunoassay Method (GH peak: ≧10 ng/ml), and the peak level of the clonidine stimulation test was 8.17 ng/ml by Radioimmunoassay Method (GH peak:≧10ng/ml), which both showed GHD. Results of karyotype analysis of peripheral blood lymphocytes showed 46, XX (Fig. 1). Subsequently, a PCR examination indicated that the SRY gene in the DNA fragment of peripheral blood lymphocytes was missing.

**Figure 2:**
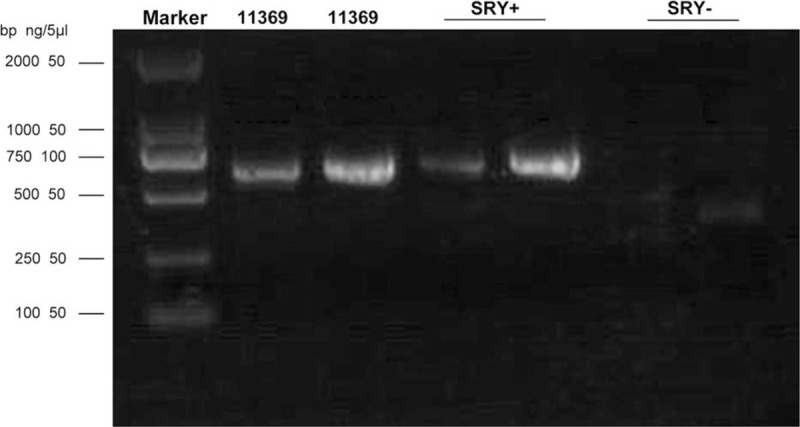
Agarose 2.0% electrophoresis: SRY gene +.

**Figure 3:**
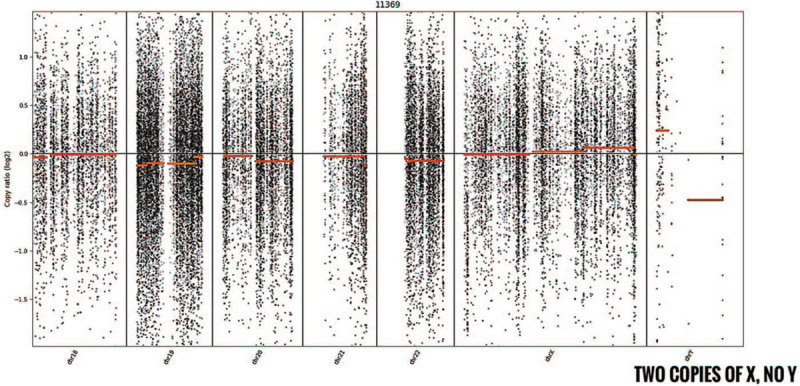
Whole exome gene sequencing detection genome copy number: 46, XX.

Based on the above test results, the patient was diagnosed with GHD and 46, XX DSD. However, considering that female gonads were not found at magnetic resonance imaging of the chest and abdomen, the whole exome gene sequencing (Exon capture) was performed (Exome V6) using the Agilent SureSelect method. Besides, high-throughput sequencing was performed on the Illumina sequencing platform. After sequencing data were analyzed by Next GENe software, mutations were screened and interpreted using the Ingenuity online software system. Candidate mutations were verified by Sanger sequencing, and the SRY gene was confirmed by electrophoresis (Fig. 2). Sequencing data revealed the presence of the SRY gene sequence and 2 copies of chromosome X (Fig. 3). In addition, no pathological variation was found in other genes. Subsequently, we used different primer sequences to carry out PCR again to detect the SRY gene, it showed a positive result. The final diagnosis was 46, XX (SRY positive) testicular DSD with GHD.

Regrettably, the patient's parents did not agree to verify the presence of the Y chromosome with multicolor fluorescent in situ hybridization, neither did they accept the treatment of recombinant human growth hormone (rhGH).

In May 2020, the boy was seven years and two months old when he came back for another visit. His height was 110.6 cm (-2.9 SDS Boys; -2.6 SDS Girls^[10]^) and BMI 14.6 kg / m^2^. This time, his testosterone level was 0.05ng/ml (reference range: < 0.1–0.35 ng/ml). Follicle-stimulating hormone was 2.26 IU/L (reference range: 0.26–3.0 IU/L), luteinizing hormone 0.03 IU/L (reference range: < 0.3 IU/L) were all within normal levels. IGFBP3 and IGF-I levels were 4.05 μg/ml (-1.3SDS 7-year-old Boys^[12]^) and 118.72 ng/ml (-1.3SDS 7-year-old Boys^[12]^) respectively, still showed low levels. Anti-Mullerian hormone was 22.65ng/ml (0–11 years old boy reference range: 38.25–332.40ng/ml ng/ml), Inhibin B was 5.23pg/ml (male reference range: 16.61–278.87 ng / ml), proved the presence of testicular tissue, but it is stunted. Besides, his bone age was delayed by 2 years (Greulich-Pyle Radiographic atlas of skeletal development of hand and wrist, Boys). We measured the GH stimulation tests again by Chemiluminescence Method. The peak level of the arginine stimulation test was 4.76 ng/ml (GH peak:≧7ng/ml^[13]^), and the peak level of the clonidine stimulation test was 5.39ng/ml (GH peak:≧7ng/ml^[13]^), showed GHD.

### 2.3 Therapeutic intervention

The boy's parents agreed to use rhGH for short stature treatment. The starting dose was 0.035 mg/kg/day, subcutaneous injection every night before going to bed. This treatment will continue until the reassessment during adolescence. We recommend testing for Y chromosome microdeletions after puberty, assessing his semen and fertility, and the low gonadal concentrations of testosterone can increase the risk of brittle fractures.

### 2.4 Follow-up and outcomes

The boy grew 2.2 cm taller within 3 months, after using rhGH treatment. He showed better intervention adherence and tolerability. But his parents still disagreed with molecular diagnostics and genomic analysis of the Y chromosome. So far, no adverse and unanticipated events have been observed.

## 3 Discussion

**Figure 4:**
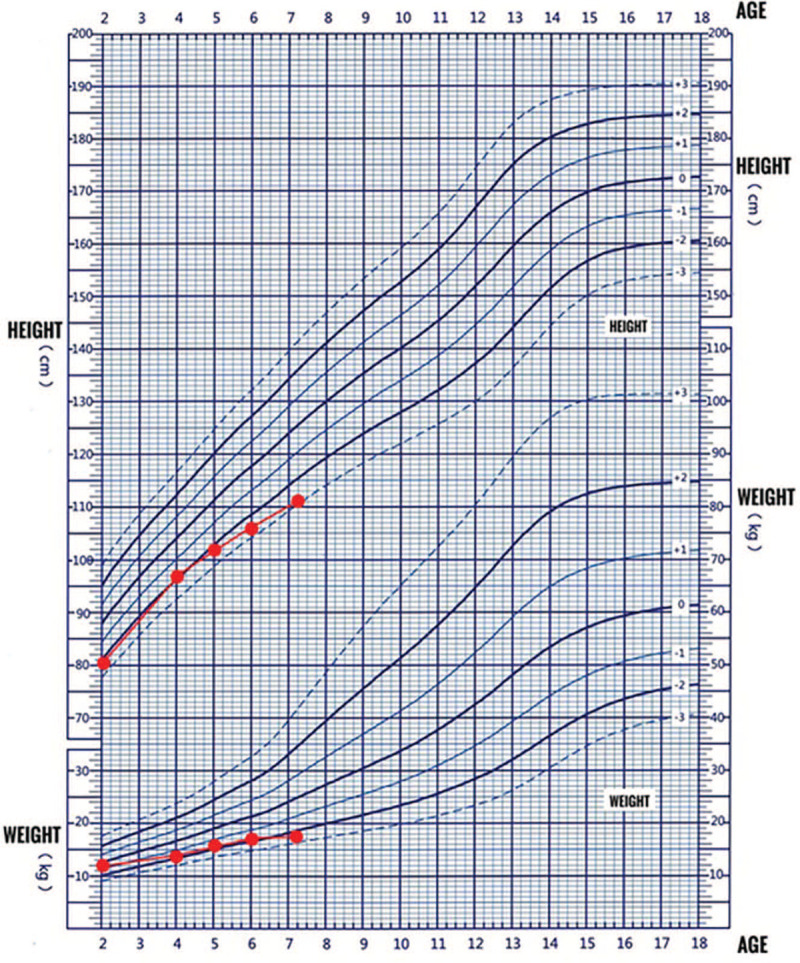
Growth curve from age 2 to present.

In this case, we believed that his short stature was originally caused by SGA at birth, but there was no catch-up growth, and the growth curve gradually deviated (Fig. 4). His bone age was delayed by 2 years, his IGFBP3 and IGF-I have always shown low levels, and GH stimulation tests performed twice showed GHD. Recent studies have shown that there is an approximately 40% incidence of GHD in SGA children needing GH treatment.^[14]^ We believed that this boy's SGA, GHD, and testicular DSD resulted in his short stature.

We tried to find the link between testicular DSD and growth failure. Previous studies have shown that the long arm of the Y chromosome contains the growth regulatory genes that control height^[15]^ and that the height of 47, XYY karyotype exceeds the average height in humans.^[16]^ Obviously, Y chromosome-Xchromosome translocation occurred in this case, resulting in the presence of SRY on the X chromosome and infertility. Thus, this boy with testicular DSD has shorter in height than age matched XY individuals. Unfortunately, the location of the Y chromosome was not confirmed in this case.

On the other hand, recent in vitro studies have identified a population of adult pituitary progenitors that express the HMG box transcription factors SOX9 and SOX2, they can become mobilized and differentiate into the appropriate endocrine cell types in response to physiological stress.^[17]^ In growth impediment induced in rats, 38% reduction of local GP ghrelin proteins and growth hormone secretagogue receptor 1a, simultaneously SRY-related transcription factor SOX9 was downregulated.^[18]^ Therefore, we believe that SOX9 and the GH / IGF1 axis work synergistically to promote body growth. However, in this case, if the SOX9 enhancer mutations resulted in 46, XX reversal and GHD, we thought that there may be an unknown connection between SOX9 and GH / IGF1 axis. It may be that the SOX9 enhancer mutations inhibits the GH / IGF1 axis, or reduces the differentiation of pituitary progenitors that express SOX9 into pituitary GH cells, resulting in growth hormone deficiency. Therefore, it is necessary to pay more attention to study the relationship between Y chromosome, SOX9 and growth hormone to treat the short stature.

Regarding the detection methods, DSD is more often detected through the etiology or histology of multistep experimental protocol. However, the diagnosis of testicular DSD is often established too late. In this case, we used at least 50 cells for karyotype analysis to avoid misdiagnosis of the 46-XX/47-XXY mosaic. Subsequently, the results of the two SRY PCR tests were completely opposite. We compared the PCR primers of these two tests and found slight differences in the 5’ and 3’ base sequence. So we thought that there must be point mutations or microdeletions in his SRY gene, which led to different results. Finally, the whole exome gene sequencing confirmed the presence of SRY gene. However, the time-consuming and costly examinations have caused the boy and his parents to reject further molecular genetic testing.

Whole exome gene sequencing is a genomic technology that interrogates the genome at a nucleotide level. It has unparalleled advantages in amplifying and capturing SRY bands, and will not be affected by point mutations or microdeletions. As we know, whole exome gene sequencing provides a diagnosis in 25–29% of individuals with disorders suggestive of a genetic etiology in pediatric and adult populations^[19]^ and it is a first-tier clinical diagnostic test for individuals with neurodevelopmental disorders.^[20]^ Therefore, in order to confirm the SRY gene in the diagnosis of testicular DSD, the accuracy of whole exome gene sequencing is markedly better than PCR. However, it is necessary to select a suitable test in accordance with the clinical manifestations of the patient so as to avoid inflicting any sort of damage to the patient.

## Author contributions

**Conceptualization:** Hanming Li.

**Investigation:** Hanming Li, Jianyu He, Iatlun Leong.

**Methodology:** Hanming Li, Jianyu He, Iatlun Leong.

**Writing – original draft:** Hanming Li.

**Writing – review & editing:** Hanming Li.

## Abbreviations

Abbreviations: DSD = disorder of sex development, GH = growth hormone, GHD = growth hormone deficiency, PCR = polymerase chain reaction, rhGH = recombinant human growth hormone, SDS = Standard Deviation Score, SGA = small for gestational age, SRY = sex determined region of Y chromosome.
